# Academic buoyancy and academic engagement in English speaking learning among Chinese college students: the mediation of enjoyment and the moderation of anxiety

**DOI:** 10.3389/fpsyg.2025.1680032

**Published:** 2025-09-15

**Authors:** Yurong Zhao, Xiaowei Huang, Lianghong Hui

**Affiliations:** Northeastern University Foreign Studies College, Shenyang, China

**Keywords:** academic buoyancy, academic engagement, foreign language enjoyment, foreign language anxiety, moderated mediation

## Abstract

**Background:**

Learners' academic engagement in English speaking learning is crucial for improving their English speaking abilities through the process of motivation. Although prior research has explored predictors of EFL (English as a Foreign Language) academic engagement, such as academic buoyancy and foreign language learning emotions, the underlying mechanisms among these variables remain largely unexplored, especially in college English speaking learning. Given this, elucidating these mechanisms is essential for advancing the understanding of how to effectively promote English speaking proficiency among college students.

**Purpose:**

This study aims to construct a structural equation model (SEM) with a sample of 244 college students from two highly-ranked Chinese universities to examine the mediating role of foreign language enjoyment and the moderating effect of foreign language anxiety in the relationship between academic buoyancy and academic engagement within the context of Chinese college English speaking learning.

**Methods:**

Based on a correlation design, data from 244 Chinese EFL college students were collected via an online questionnaire in December 2023 and analyzed through a moderated mediation model with SPSS 26.0, IBM Amos 22, Mplus 8.3, and PROCESS v4.2, applying 2,000 bootstrap iterations.

**Results:**

The results reveal that English speaking learning buoyancy (ESLB) positively predicted English speaking learning engagement (ESLEG) both directly and indirectly through the mediating role of English speaking learning enjoyment (ESLE). Additionally, English speaking learning anxiety (ESLA) significantly and negatively moderated the relationships between ESLB and ESLE, as well as between ESLB and ESLEG.

**Conclusion:**

These findings highlight the complex interactions of academic buoyancy and foreign language learning emotions on engagement in English speaking learning. The study provides valuable pedagogical implications for enhancing English speaking instruction in Chinese colleges.

## 1 Introduction

Chinese EFL students often struggle with limited spoken English capacity, a critical aspect of their overall linguistic proficiency (Xing and Bolden, 2019). Developing this capacity, is a long-term process heavily shaped by learning motivation (Wang, 2017), which operates within a complex dynamic system of various factors strongly influencing each other (Xu and Wang, 2025). For example, academic buoyancy enables EFL learners to effectively manage academic setbacks, directly influencing their learning motivation (Derakhshan et al., 2025) and significantly predicting learning engagement (Yang and Du, 2023). Furthermore, academic emotions in foreign language learning, such as enjoyment, are strongly associated with learning motivation (Jia and Cheng, 2022), which in turn predicts learning engagement (Yang and Du, 2023). Academic engagement is essential for enhancing English speaking performance and achievement (Skinner and Pitzer, 2012; Truta et al., 2018). However, while many studies have examined the predictors of academic engagement (Merhi et al., 2018; Oriol-Granado et al., 2017; Woreta, 2024), the dynamic interplay among academic engagement, academic buoyancy, and foreign language learning emotions, particularly in the context of English speaking learning, remains underexplored. Given this gap, investigating the underlying mechanisms connecting these variables among Chinese EFL college students is crucial to advance our understanding of their collective impact on English speaking learning.

Academic buoyancy predicts engagement (Wang, 2024) and mitigates adversity in language learning (Martin and Marsh, 2020). However, its role in English speaking learning among Chinese college students is understudied (Wang and Liu, 2022; Liu et al., 2025). Chinese college students frequently encounter both internal and external setbacks hindering their academic performance in the process of English speaking learning. Therefore, it is crucial to examine the predictive role of English speaking learning buoyancy in relation to English speaking learning engagement (Xiao, 2012), especially among Chinese college students.

Emotional factors play a pivotal role in language acquisition (Krashen, 1982; Liu and Li, 2022). Recent studies have integrated Krashen's hypothesis to show the distinct impacts of emotions on English speaking learning (Lemana et al., 2023; Lou, 2015). Some research has also delved into the application of positive psychology in diverse academic settings (Okur et al., 2023), especially in second language acquisition (SLA), highlighting the facilitating roles of positive emotions, including enjoyment, and the impeding effects of negative emotions, such as anxiety (Ge, 2023; Wang et al., 2023). However, the existing research focuses more on the respective roles of enjoyment and anxiety on engagement (Tsang and Dewaele, 2024), while ignoring the interactive role of both enjoyment and anxiety on engagement, especially in the context of college students' English speaking learning.

This study aims to construct a structural equation model (SEM) with 244 college students to explore the mediating role of foreign language enjoyment and the moderating effect of foreign language anxiety between academic buoyancy and academic engagement in English speaking learning. This study endeavors to advance the application of positive psychology in the English speaking learning context and to offer practical pedagogical insights that can contribute to future teaching and learning in this area.

## 2 Literature review

### 2.1 Academic engagement

Engagement has garnered substantial attention in both work and school contexts (Salmela-Aro and Upadaya, 2012). Workplace engagement is characterized by three core dimensions: energy, dedication, and absorption (Schaufeli et al., 2002). Specifically, energy reflects mental resilience, dedication denotes commitment and responsibility, while absorption indicates deep focus (Salmela-Aro and Upadaya, 2012).

In classrooms, engagement is dynamic and contextualized, typically conceptualized as behavioral (paralleling absorption), cognitive (corresponding to dedication), agentic or emotional (akin to energy or vigor), and social engagement, especially significant in language classrooms (Salmela-Aro and Upadaya, 2012; Hoi and Hang, 2021; Hiver et al., 2021; Luo et al., 2022). However, some researchers focus on energy, dedication, and absorption as core dimensions of academic engagement to preserve its continuity and coherence (Salmela-Aro and Upadaya, 2012). This study aligns with (Schaufeli et al. 2002) to measure the most primitive and authentic level of academic engagement. Academic engagement in this study is described as absorption, energy, and dedication within the context of English speaking learning. Specifically, absorption refers to a high degree of focus on the English speaking classroom, to the point of ignoring time; energy denotes a proactive strategy and pathway toward English speaking learning; and dedication represents a sincere and genuine perception of the significance of English speaking learning.

In foreign language learning, mastering proficient spoken English is highly significant (Lee et al., 2020). Research consistently shows that engagement positively impacts EFL achievement and performance (Wang et al., 2023; Nugiel et al., 2023). Specifically, engagement in English speaking learning is a significant predictor of English speaking performance (Wang et al., 2024). Given its crucial role, exploring factors influencing English speaking learning engagement is essential.

### 2.2 Academic buoyancy

Academic buoyancy is defined as the capacity to navigate academic setbacks and challenges, such as negative feedback and difficulties in interactions with peers and teachers (Martin and Marsh, 2008; Putwain and Wood, 2023). Learners with higher academic buoyancy are better able to deal with learning adversities and are more likely to expend greater effort and energy to achieve their goals (Yang and Liang, 2025).

In the EFL context, the positive role of academic buoyancy in English learning has been extensively examined. (Jia and Cheng 2022) demonstrated that academic buoyancy, along with social support, predicted English motivation among undergraduates. (Xu and Wang 2024) discovered that academic buoyancy could predict self-regulated writing strategies mediated by both positive and negative academic emotions. (Li et al. 2023) uncovered that academic buoyancy fully mediated the relationship between teachers' support and educational outcomes. (Fu 2024) further explored the moderating role of academic buoyancy on the path from social support to learning burnout.

Moreover, the positive association between academic buoyancy and academic engagement has been well-documented across different age groups and educational contexts. For instance, (Bostwick et al. 2022) studied secondary school students (Grades 7–11) in Australia, revealing a directional pathway from academic buoyancy to students' engagement. (Putwain and Wood 2023) examined primary school students in mathematics learning, identifying a similar path. Additionally, (Liu et al. 2024) verified the indirect protective role of academic buoyancy on EFL engagement. In a similar vein, (Han and Eerdemutu 2025) found that among Chinese students learning Japanese, academic buoyancy also positively impacted engagement indirectly.

**Figure 1:**
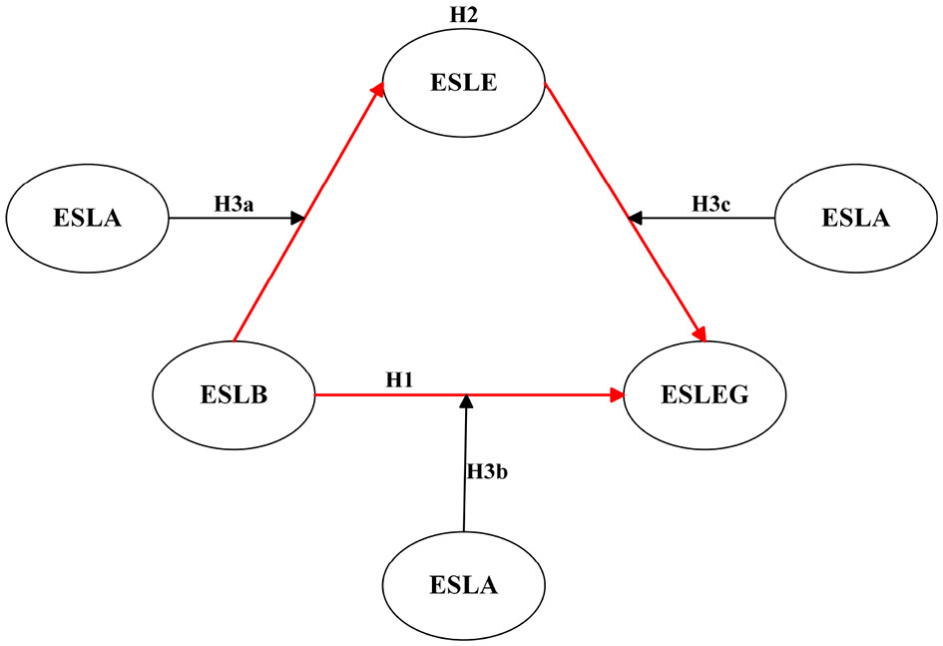
Hypothesized model. *N* = 244. ESLB, English speaking learning buoyancy; ESLE, English speaking learning enjoyment; ESLEG, English speaking learning engagement; ESLA, English speaking learning anxiety.

Building on this foundation, the present research further examines the predictive role of English speaking learning buoyancy (ESLB) on English speaking learning engagement (ESLEG) and proposes the first hypothesis (see Figure 1).

H1. ESLB significantly and positively predicts ESLEG.

### 2.3 Foreign language enjoyment

English enjoyment, considered “an emotional key to unlocking learners' potential” (Dewaele and MacIntyre, 2014; p. 261), is related to academic buoyancy (Hirvonen et al., 2020). According to Pekrun's (2006) control-value theory, emotions are dynamic processes involving multiple psychological subsystems, such as affect and cognition. Specifically, achievement emotions, categorized into activity and outcome emotions, provide a comprehensive framework for analyzing the emotions experienced in academic and achievement contexts. In this study, foreign language enjoyment, arising from classroom learning, was categorized as a positive activity emotion. Additionally, Fredrickson's (2003) broaden-and-build theory suggests that positive emotions expand an individual's immediate range of cognitive and behavioral responses, fostering the development of lasting personal resources and enhancing academic engagement. Collectively, these theories provide a foundation for understanding the interrelationships among academic buoyancy, enjoyment, and academic engagement.

The significant relationship between enjoyment and academic buoyancy has been well-established in prior research. (Zhou and Wang 2024) revealed that academic buoyancy moderated the relationship between enjoyment and test performance among 563 Chinese college English learners. In online learning, (Wang and Hui 2024) confirmed the predictive role of buoyancy on enjoyment within 442 Chinese EFL learners. However, the direct relationship between academic buoyancy and enjoyment in college English speaking classes remains underexplored.

Consistent with the theoretical frameworks discussed above, prior studies have demonstrated that enjoyment enhanced academic engagement (Liu, 2022; Wang, 2022). For example, (Zhang et al. 2024) found that enjoyment significantly supported engagement among junior high EFL learners, with stronger effects than burnout. Similarly, (Derakhshan and Noughabi 2024) identified foreign language enjoyment as the strongest predictor of engagement among EFL learners aged 18 to 48. Therefore, enjoyment likely significantly and positively predicts English speaking learning engagement.

Building on previous literature on the positive relationship between enjoyment and academic buoyancy with engagement, this study tests the mediating role of English speaking learning enjoyment (ESLE) between English speaking learning buoyancy (ESLB) and English speaking learning engagement (ESLEG). Given the established positively predictive effect of academic buoyancy on enjoyment and the documented impact of enjoyment on academic engagement, this study logically proposes the second hypothesis (see Figure 1).

H2. ESLE significantly and positively mediates the relationship between ESLB and ESLEG.

### 2.4 Foreign language anxiety

Speaking activities often trigger the highest levels of anxiety in second/foreign language activities (Liu and Hong, 2021). Anxiety, defined as a personal experience characterized by tension, apprehension, nervousness, and worry, has been verified to significantly hinder learners' performance in foreign language classes (Horwitz et al., 1986). Recent studies have explored and expanded the concept of xenophonophobia, defined as the fear of all foreign sounds, to investigate the intersection of foreign language learning and multiculture or advanced deep learning models. These studies aim to foster a sense of belonging and reduce foreign language speaking anxiety from innovative perspectives (Lisiak et al., 2021; Park et al., 2022).

Lately, there has been growing interest in the association between anxiety and enjoyment (Fang and Tang, 2021). Related studies have found that participants' levels of enjoyment were significantly higher than their anxiety levels, with enjoyment being perceived more frequently, as evidenced by data collected from Chinese English majors (Fang and Tang, 2021). Furthermore, (Aubrey 2022) observed a negative relationship between these two emotions during second language speaking tasks. This finding was further supported by (Li 2024), who found a significantly negative correlation between enjoyment and anxiety among Chinese junior high school students in foreign language learning. More recently, Durmuş and Kiziltan (2025) also discovered a significant negative correlation between enjoyment and anxiety among freshmen in online speaking skills courses. Collectively, these studies provide consistent evidence that anxiety and enjoyment are inversely related in foreign language learning. In order to further explore the relationship between foreign language anxiety and enjoyment among Chinese undergraduate English majors, (Li 2025) revealed that foreign language anxiety significantly and negatively correlated with all three sources of foreign language enjoyment, namely FLE-private, FLE-atmosphere, and FLE-teacher. Moreover, recent pioneering studies have explored the interaction between enjoyment and anxiety in influencing EFL engagement, revealing a positive association between the interplay of these emotions and emotional or cognitive engagement (Wang et al., 2025). These findings highlight the dynamic relationship between positive and negative emotions in foreign language learning contexts, underscoring the need for further investigation into how anxiety influences enjoyment and how their interplay shapes academic engagement, particularly in English speaking learning environments.

Moreover, the relationship between anxiety and academic engagement is complex, with recent studies presenting multifaceted effects. (Ng et al. 2022) have demonstrated the negative effects of higher anxiety on academic engagement among Australian students. Similarly, (Wang et al. 2023) found that negative emotions like foreign language anxiety would narrow learners' learning behaviors, thereby limiting their willingness to participate in foreign language learning. In contrast, (Liu et al. 2025) proposed that test-focused Chinese EFL learners may invest more effort due to anxiety, suggesting anxiety could positively predict behavioral engagement in some contexts. Similarly, the positive relationship between anxiety and engagement was also reported by (Yang and Rui 2025), who found that EFL students with higher anxiety inclined to engage more, corroborating the importance of emotions on student engagement. These conflicting findings highlight the need for further exploration of the specific correlation between English speaking learning anxiety and engagement.

Furthermore, prior research has begun to explore the interplay between anxiety and academic buoyancy in various academic settings. However, the specific mechanisms of this interaction have not been thoroughly investigated, despite increasing recognition of the importance of understanding how anxiety and buoyancy interacted to influence academic performance. For instance, (Putwain et al. 2022) found that academic buoyancy moderated the association between anxiety and test performance, suggesting an interactive impact of academic buoyancy and anxiety on test performance. In the EFL context, enjoyment—one of the main positive achievement emotions—has been shown to be positively related to EFL performance (Yu et al., 2022). Additionally, agentic engagement—one dimension of academic engagement—has been found to have a fully mediating effect on EFL test performance (Eren and Rakicioğlu-Söylemez, 2023). Building on these findings and guided by Fredrickson's (2003) broaden-and-build theory, it is reasonable to assume that academic buoyancy and anxiety may interact to shape EFL learners' emotional (e.g., enjoyment) and behavioral (e.g., academic engagement) responses, subsequently affecting test performance. Specifically, anxiety may play a crucial role in shaping how academic buoyancy influences learners' emotional and behavioral responses in EFL contexts.

Based on the close interrelationships among foreign language anxiety, academic buoyancy, foreign language enjoyment, and academic engagement, this study logically proposes that English speaking learning anxiety (ESLA) may moderate the three sub-paths on the nexus from ESLB to ESLEG via ESLE, as presented in Figure 1. However, given the multifaceted effects of anxiety on enjoyment and academic engagement in EFL learning, the direction and strength of its moderating role on the nexus from ESLB to ESLEG via ESLE require further empirical investigation.

H3a. ESLA positively or negatively significantly moderates the relationship between ESLB and ESLE.
H3b. ESLA positively or negatively significantly moderates the relationship between ESLB and ESLEG.
H3c. ESLA positively or negatively significantly moderates the relationship between ESLB and ESLEG via ESLE.

### 2.5 The present study

To bridge the gap in previous research and address existing issues regarding the correlations among academic buoyancy, academic engagement, foreign language enjoyment, and foreign language anxiety, particularly in the context of college students' English speaking learning, the present study employs structural equation modeling to construct a moderated mediation model. This model conceptualizes the complex and nuanced relationships among these variables. Specifically, this study aims to advance understanding in this area by addressing the following three research questions.

RQ1: Does ESLB significantly predict ESLEG?
RQ2: Does ESLE mediate the relationship between ESLB and ESLEG?
RQ3: Does ESLA moderate the relationships among ESLB, ESLE, and ESLEG?

## 3 Method

### 3.1 Participants

This study was conducted with 244 Chinese students who voluntarily participated from English speaking courses at two highly-ranked universities. One is a top-tier comprehensive university in Beijing, renowned for its outstanding academic reputation and research strength across various fields. The other is a comprehensive university in Hebei Province, China, with a strong emphasis on engineering and technology. The sample included 137 males (56.1%) and 107 females (43.9%), representing four academic levels: 28 freshmen (11.4%), 166 sophomores (68.0%), 19 graduate students (7.7%), and 31 doctoral candidates (12.7%). They were enrolled in various fields of study, including 120 in science and engineering (49.1%), 89 in economics and management (36.4%), 29 in foreign languages (11.8%), and 6 in literature and history (2.4%). Participants had been learning English for over 9 years. In a self-assessment of their English speaking proficiency on a 7-point scale (1 = worst, 7 = best), 52.7% scored themselves as below-medium level (scores 1–3), and 46.9% as above-medium level (scores 4–7). This diverse sample broadens the generalizability of the research conclusions.

### 3.2 Measures

This study employed a comprehensive questionnaire encompassing multiple sections (see Supplementary Table S1). Three experienced experts adapted the words of four well-established scales to align with the context of this study, ensuring that each item was tailored to reflect the English-speaking learning experiences of the participants while retaining the English language version. The first section collected demographic information of the participants, as presented in the previous contents. And the second section focused on participants' experiences during the process of English speaking learning, specifically measuring their levels of buoyancy, enjoyment, engagement, and anxiety. Responses were rated on a 7-point Likert scale, with 1 indicating strong disagreement and 7 indicating strong agreement. The four measures are as follows.

#### 3.2.1 English speaking learning buoyancy scale

This scale was adapted from the questionnaire developed by (Martin and Marsh 2008), with modifications to assess academic buoyancy specifically in the context of English speaking learning. Compared with the original scale, all the four items in the adapted scale addressed setbacks, pressure, and confidence in English speaking learning, such as “I can deal with pressure in English speaking learning.” After conducting confirmatory factor analysis (CFA) and testing for internal consistency, the scale demonstrated excellent reliability (Cronbach's α = 0.909>0.8) and construct validity, with the following fit indices: χ^2^/df = 1.665 (< 3), CFI = 0.999 (>0.95), TLI = 0.996 (>0.95), RMSEA = 0.046 (< 0.06), and SRMR = 0.01 (< 0.08) (Hu and Bentler, 1999; Wang, 2014).

#### 3.2.2 English speaking learning enjoyment scale

The scale applied in this study was a short form of the Foreign Language Enjoyment Scale (Botes et al., 2021), which was adapted to reflect experiences in English speaking learning. These adapted questions were oriented to both inside and outside the English speaking classroom and consisted of six items, with a sample item being “The English speaking teacher always encourages us.” The scale demonstrated acceptable reliability (Cronbach's α = 0.883>0.8). Construct validity was assessed by CFA, yielding the following fit indices: χ^2^/df = 2.346 (< 3), CFI = 0.997 (>0.95), TLI = 0.989 > (0.95), RMSEA = 0.065, and SRMR = 0.02 (< 0.08) (Hu and Bentler, 1999; Wang, 2014).

#### 3.2.3 English speaking learning engagement scale

The EDA scale, proposed by (Salmela-Aro and Upadaya 2012), firstly measures key components of schoolwork engagement. Therefore, our study adapted it to the context of English speaking learning and captured the most direct and primitive condition of participants' English speaking learning engagement. The adapted scale consisted of nine items and considered three key dimensions: energy, dedication, and absorption when measuring English speaking learning engagement. A sample item is, “I am filled with energy in English speaking learning.” The scale showed excellent reliability (Cronbach's α = 0.942>0.8) and strong construct validity (χ^2^/df = 1.324 < 3, CFI = 0.998>0.95, TLI = 0.996>0.95, RMSEA = 0.032 < 0.06, and SRMR = 0.015 < 0.08) (Hu and Bentler, 1999; Wang, 2014).

#### 3.2.4 English speaking learning anxiety scale

This scale was derived from the Foreign Language Classroom Anxiety Scale (FLCAS) developed by (Botes et al. 2022) and adapted to the context of English speaking learning. Eight items were refined to assess the level of anxiety that participants experienced during English speaking classes and when listening to their classmates speak English, emphasizing the unique features of spoken English learning, such as interaction-related pressure. A sample item is “I always think that my classmates speak English better than me.” The scale demonstrated acceptable internal consistency (Cronbach's α = 0.881>0.8) and excellent construct validity (χ^2^/df = 1.697 < 3, CFI = 0.991>0.95, TLI = 0.983>0.95, RMSEA = 0.047 < 0.06, and SRMR = 0.027 < 0.08) (Hu and Bentler, 1999; Wang, 2014).

### 3.3 Data collection

The data for this study were collected via an online questionnaire platform, “Wenjuanxing.” Prior to data collection, English speaking teachers of the participants were contacted to ensure voluntary consent from both teachers and students. The participants in this study were Chinese students; thus, the adapted scales used in the questionnaire were translated into Chinese, followed by back-translation to ensure accuracy and fidelity, with expert review to verify the translations. To ensure construct equivalence, the construct validity and reliability of all scales were assessed, with statistical results reported above. Additionally, a pre-test was conducted with six experts and teachers to ensure the scales' readability and comprehensibility. With consent obtained and study requirements explained, the questionnaires were distributed by the participants' English speaking teachers during their regular classes, from December 12th to 30th, 2023. Out of 315 distributed questionnaires, 244 valid responses were received, with incomplete or duplicate responses excluded. All data were anonymized to protect the privacy of the participants.

### 3.4 Data analysis

In this research, four analytical tools were employed to test and analyze the data. To begin with, the construct validity of all the scales in the questionnaire was assessed via confirmatory factor analysis (CFA) in IBM Amos 22. Then the reliability of all the scales was examined by SPSS 26.0. Additionally, SPSS 26.0 was utilized for descriptive analyses, calculating the mean, standard deviation, skewness, and kurtosis of the collected data. Subsequently, a correlational analysis was conducted to explore the potential relationships among involved variables.

Next, the SEM was analyzed in Mplus 8.3 to assess the direct relationship between ESLB and ESLEG, as well as the statistical significance of the mediating effect of ESLE between ESLB and ESLEG. Bootstrapping with 2,000 resampling iterations was performed to confirm the mediating effect. Finally, PROCESS v4.2 was applied to verify the moderating effect of ESLA on the entire pathway from ESLB to ESLEG via ESLE. The following section presents the statistical results of this study.

## 4 Results

### 4.1 Descriptive analysis

Based on the descriptive analysis conducted using SPSS 26.0 (see Table 1), the levels of academic buoyancy, enjoyment, engagement, and anxiety in the context of English speaking learning were measured, respectively. The results indicated that, generally, the participants exhibited relatively high levels of English speaking learning buoyancy, enjoyment, and engagement. In contrast, their English speaking learning anxiety was at an above-average level. According to the correlational results, the target variables mentioned above were significantly correlated with each other (see Table 1).

**Table 1:** Results of descriptive analysis and correlations of measures for ESLB, ESLE, ESLEG, and ESLA.

**Variable**	**Mean**	**SD**	**Skewness**	**Kurtosis**	**1**	**2**	**3**	**4**
ESLB	5.015	1.069	−0.184	−0.054	1.000			
ESLE	5.947	0.761	−0.774	0.050	0.388**	1.000		
ESLEG	5.186	0.922	0.241	−0.701	0.564**	0.659**	1.000	
ESLA	3.883	1.155	0.027	−0.116	−0.542**	−0.225**	−0.366**	1.000

*N* = 244, **p < 0.01. ESLB, English speaking learning buoyancy; ESLE, English speaking learning enjoyment; ESLEG, English speaking learning engagement; ESLA, English speaking learning anxiety.

### 4.2 Structural equation model (SEM)

Before analyzing the structural equation model, a general goodness-of-fit test was developed, whose results indicated that this model reached an acceptable data fit (χ^2^/df = 2.135 < 3, RMSEA = 0.068, CFI = 0.966>0.95, TLI = 0.957>0.95, and SRMR = 0.103) (Hu and Bentler, 1999; Wang, 2014).

**Figure 2:**
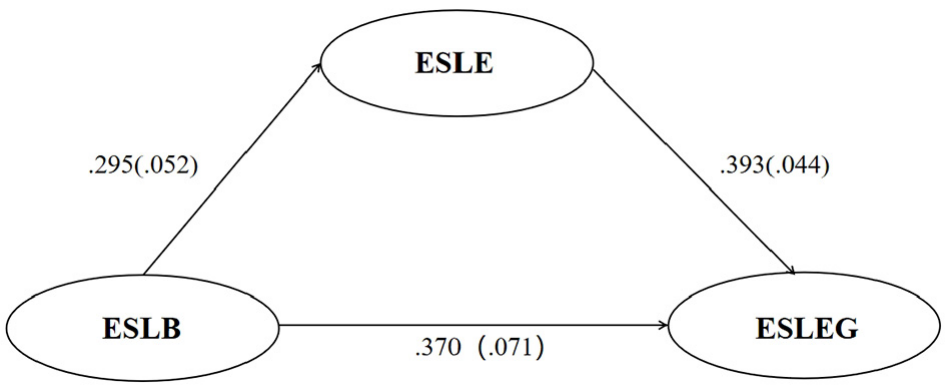
The standardized results of the statistical diagram of the hypothesized model in Mplus 8.3. *N* = 244. ESLB, English speaking learning buoyancy; ESLE, English speaking learning enjoyment; ESLEG, English speaking learning engagement.

Following this, the analysis of this model was developed. As shown in Figure 2 and Table 2, ESLB was found to be a significant positive predictor of ESLE (β = 0.295, *p* < 0.001), and ESLE significantly and positively predicted ESLEG (β = 0.393, *p* < 0.001). Additionally, a significant positive correlation was observed in the direct path from ESLB to ESLEG (β = 0.370, *p* < 0.001). These findings addressed RQ1 and provided support for H1.

**Table 2:** The standardized results of the direct and indirect path coefficients of the hypothesized model in Mplus 8.3.

**Path**	**Estimate (β)**	**SE**	**Est./SE**	**p**	**95% Confidence interval**	
					**Lower 5%**	**Upper 5%**
ESLB → ESLE	0.295	0.052	5.680	< 0.001	–	–
ESLE → ESLEG	0.393	0.044	8.870	< 0.001	–	–
ESLB → ESLEG	0.370	0.071	5.232	< 0.001	–	–
ESLB → ESLE → ESLEG (indirect)	0.116	0.026	4.483	< 0.001	0.078	0.165

*N* = 244. ESLB, English speaking learning buoyancy; ESLE, English speaking learning enjoyment; ESLEG, English speaking learning engagement.

#### 4.2.1 The results of the mediating effect of ESLE

Next, a further test using bootstrapping with 2000 samples was conducted. The yielded results (see Table 2) indicated that ESLE significantly and positively mediated the relationship between ESLB and ESLEG (β = 0.116, *p* < 0.001; 95% CI = 0.078 to 0.165). Given that the direct effect of ESLB on ESLEG was also statistically significant (β = 0.370, *p* < 0.001), it can be concluded that ESLE acted as a partial mediator between ESLB and ESLEG. These findings addressed RQ2 and provided support for H2.

#### 4.2.2 The results of the moderating effect of ESLA

**Figure 3:**
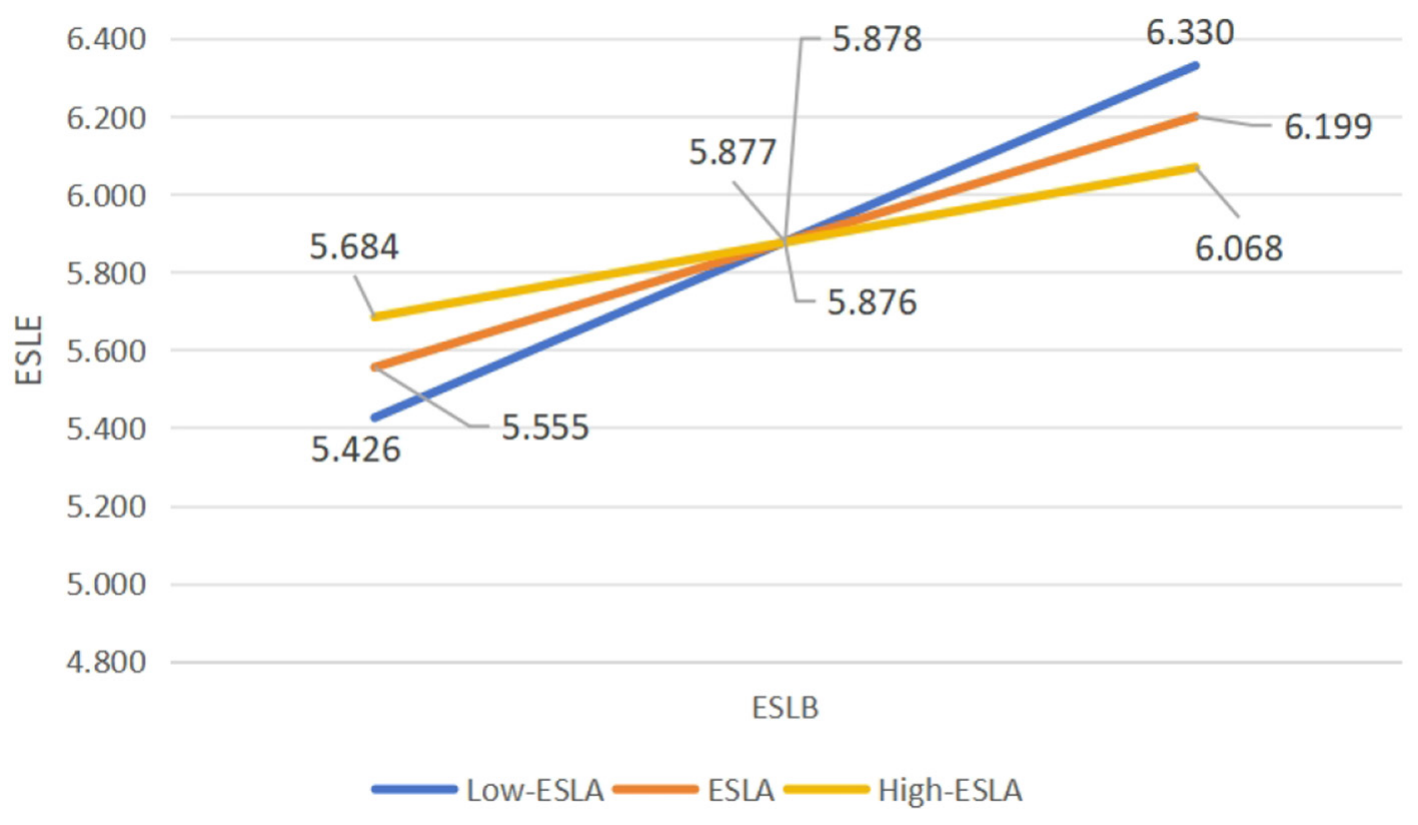
The moderation pattern of ESLA on the slope between ESLB and ESLE in the hypothesized model. *N* = 244. ESLA, English speaking learning anxiety; ESLB, English speaking learning buoyancy; ESLE, English speaking learning enjoyment.

As shown in Table 3, the interaction of the sub-path ESLB × ESLA on ESLE was significantly different from zero (β = −0.105, *p* < 0.01; 95% CI = −0.167 to −0.044). The results indicated that ESLA significantly and negatively moderated the relationship between ESLB and ESLE. They addressed RQ3 and hereby substantiated H3a. The moderation pattern of ESLA on the slope between ESLB and ESLE is illustrated in Figure 3.

**Table 3:** The statistical results of the moderating effects of ESLA in the hypothesized model.

**Variable**	**Parametric estimation**		**t**	**p**	**95% Bootstrap**	
	**Coeff (**β**)**	**SE**			**LLCI**	**ULCI**
Int-1	−0.105	0.031	−3.380	0.001	−0.167	−0.044
(ESLB*ESLA → ESLE)						
Int-1	−0.101	0.044	−2.302	0.022	−0.187	−0.015
(ESLB*ESLA → ESLEG)						
Int-1	0.009	0.058	0.162	0.871	−0.105	0.124
(ESLE*ESLA → ESLEG)						

*N* = 244. ESLB, English speaking learning buoyancy; ESLE, English speaking learning enjoyment; ESLEG, English speaking learning engagement; ESLA, English speaking learning anxiety.

To clarify the conditional indirect effects of the moderator variable ESLA, its impact was tested at different values (the mean and ±1SD). As shown in Table 4, the moderating effect of ESLA on the relationship between ESLB and ESLE was significant across all three levels: low (β = 0.423, *p* < 0.001), mean (β = 0.301, *p* < 0.001), and high (β = 0.180, *p* < 0.01). Notably, the moderating coefficient was highest at the low level of ESLA. These findings indicated that ESLA moderated the sub-path from ESLB to ESLE at all three levels, with the strongest positive predictive effect of ESLB on ESLE occurring when learners experienced low anxiety.

**Table 4:** The moderating coefficients of ESLA on the sub-path from ESLB to ESLE in the hypothesized model.

**Moderator**	**Parametric estimation**		**t**	**p**	**95% Bootstrap**	
**ESLA**	**Effect (**β**)**	**SE(HC3)**			**LLCI**	**ULCI**
−1.155	0.423	0.074	5.739	< 0.001	0.278	0.568
0.000	0.301	0.056	5.427	< 0.001	0.192	0.411
1.155	0.180	0.058	3.116	0.002	0.066	0.293

*N* = 244. ESLA, English speaking learning anxiety; ESLB, English speaking learning buoyancy; ESLE, English speaking learning enjoyment.

**Figure 4:**
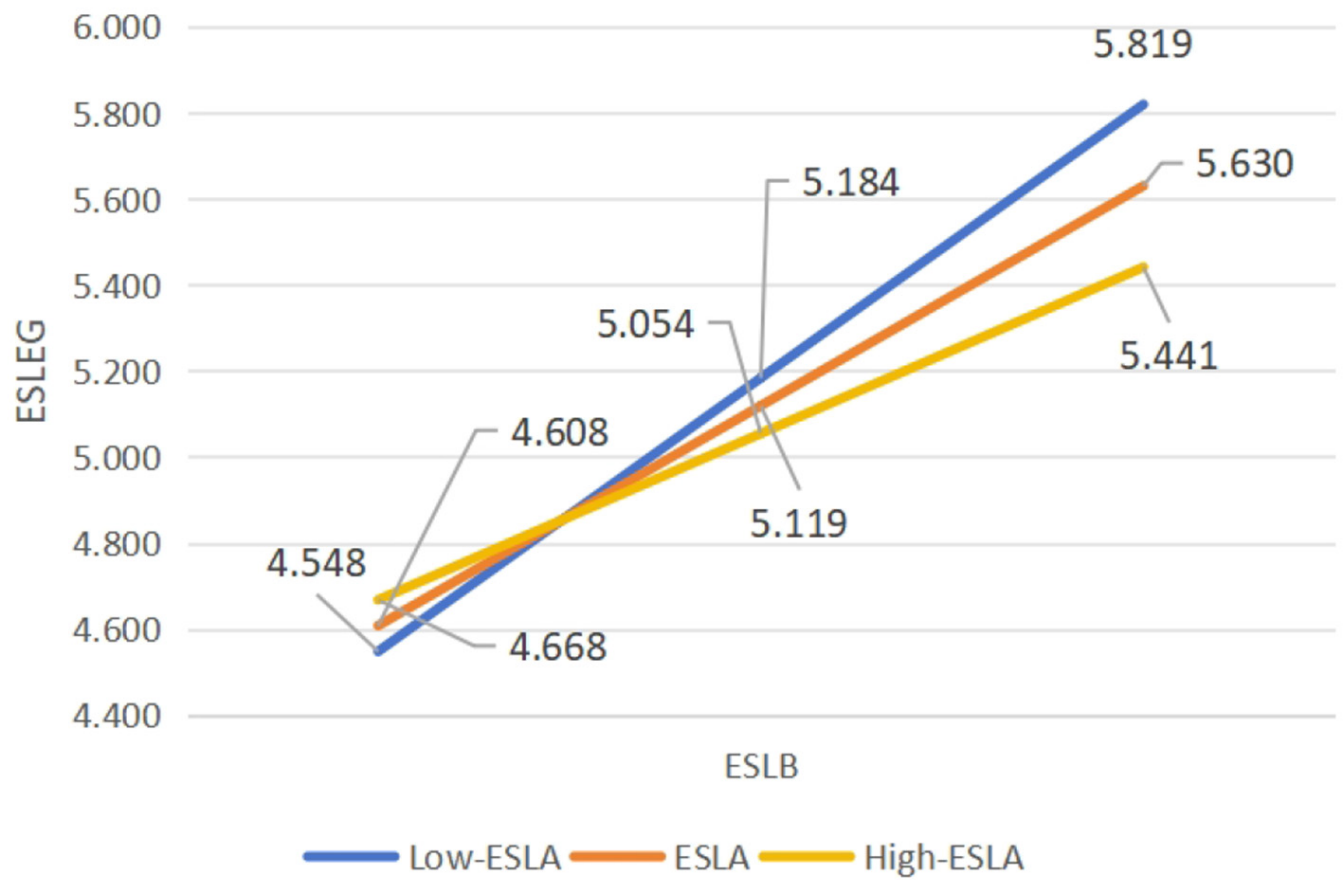
The moderation pattern of ESLA on the slope between ESLB and ESLEG in the hypothesized model. *N* = 244. ESLA, English speaking learning anxiety; ESLB, English speaking learning buoyancy; ESLEG, English speaking learning engagement.

In addition, the interaction of the sub-path ESLB × ESLA on ESLEG was also significantly different from zero (β = −0.101, *p* < 0.05; 95% CI = −0.187 to −0.015) (refer to Table 3). Therefore, H3b was substantiated. Figure 4 depicts the pattern of moderation of ESLA on the slope between ESLB and ESLEG.

Finally, the region of the conditional indirect effect of ESLA on ESLB and ESLEG was examined, and it was found that the moderating effect was also significant across all three levels: low (β = 0.595, *p* < 0.001), mean (β = 0.478, *p* < 0.001) and high (β = 0.362, *p* < 0.001) (see Table 5). Specifically, the moderating effect was strongest at the low level of ESLA, with the highest moderating coefficient in this case. This suggested that the prediction of ESLEG by ESLB was the strongest when learners experienced low anxiety.

**Table 5:** The moderating coefficients of ESLA on the sub-path from ESLB to ESLEG in the hypothesized model.

**Moderator**	**Parametric estimation**		***t***	***p***	**95% Bootstrap**	
**ESLA**	**Effect (**β**)**	**SE(HC3)**			**LLCI**	**ULCI**
−1.155	0.595	0.083	7.211	< 0.001	0.432	0.757
0.000	0.478	0.070	6.861	< 0.001	0.341	0.616
1.155	0.362	0.090	4.035	< 0.001	0.185	0.538

*N* = 244. ESLA, English speaking learning anxiety; ESLB, English speaking learning buoyancy; ESLEG, English speaking learning engagement.

However, the interaction effect of the sub-path ESLE*ESLA on ESLEG was not statistically significant (β = 0.009, *p* = 0.871; 95% CI = −0.105 to 0.124) (see Table 3). Therefore, H3c was not supported.

## 5 Discussion

This study examined the relationship between academic buoyancy and engagement in college English speaking learning, as well as the interactive mechanisms involving foreign language enjoyment and anxiety, using a structural equation modeling (SEM) approach. It indicated that academic buoyancy directly and indirectly predicted academic engagement in English speaking learning through the mediating role of enjoyment. Furthermore, anxiety was found to moderate the relationships between academic buoyancy and enjoyment, as well as between academic buoyancy and engagement. The findings of the current study are discussed in the following three sections.

### 5.1 The direct effect of English speaking learning buoyancy (ESLB) on engagement (ESLEG)

The study showed that academic buoyancy significantly predicted academic engagement in English speaking learning (β = 0.370, *p* < 0.001). The statistical result is similar to (Zhai 2025), who found that academic buoyancy significantly predicted academic engagement (β = 0.40, *p* < 0.001), suggesting that academic buoyancy had a moderately low but significantly predictive effect on academic engagement. In this study, learners who were better at dealing with setbacks, pressure, and stress, and maintained mental stability in spoken English learning—such as those who agreed with items on the English Speaking Learning Buoyancy Scale like “I am good at dealing with setbacks in spoken English learning (e.g., poor grades, negative feedback on my spoken English)” and “I do not let a poor spoken English score affect my confidence,”—exhibited greater energy, dedication, and absorption in their learning process. These learners displayed heightened energy, perceived spoken English learning as meaningful, and focused on skill improvement, as captured by items on the English Speaking Learning Engagement Scale, such as “I find learning spoken English to be meaningful” and “I feel energetic when learning spoken English.” This finding aligns with prior research on the positive relationship between academic buoyancy and engagement in different educational contexts (Bostwick et al., 2022; Putwain and Wood, 2023; Liu et al., 2024; Han and Eerdemutu, 2025). The predictive role of ESLB on ESLEG may be explained by the influence of autonomy-supportive climates on learners' engagement in English speaking classrooms (Dincer et al., 2012). Moreover, this finding aligns with the established logic that flipped classrooms can enhance learners' engagement in English speaking learning, which requires learners to be proactive and tackle challenges independently (Wang and Wang, 2017; Li and Li, 2022). Therefore, academic buoyancy may serve as a direct and significant predictor in prompting academic engagement in English speaking learning contexts.

### 5.2 The mediating effect of English speaking learning enjoyment (ESLE) between ESLB and ESLEG

Additionally, the study revealed that academic buoyancy significantly predicted engagement via the mediation of enjoyment in English speaking learning (β = 0.116, *p* < 0.001; 95% CI = 0.078 to 0.165), aligning with (Hirvonen et al. 2020), who suggested that buoyancy fostered a positive emotional environment to enhance engagement. First, academic buoyancy significantly predicted enjoyment (β = 0.295, *p* < 0.001). Learners who effectively managed stress and maintained positive attitudes in English speaking learning, as indicated by their agreement with items on the English Speaking Learning Buoyancy Scale like “I can deal with pressure in English speaking learning” and “I am skilled at managing academic stress related to spoken English learning,” perceived more enjoyment. This enjoyment was characterized by positive emotional experiences, supportive interactions with friendly teachers and interesting teaching contents, and joyful classroom atmospheres, as reflected in items on the English Speaking Learning Enjoyment Scale, such as “I enjoy learning spoken English,” “The English speaking teacher is very friendly,” and “There is a lot of laughter and joy in the spoken English class.” This finding extends (Xu and Wang 2024), who identified a significant correlation between academic buoyancy and positive academic emotions, such as enjoyment, showing that academic buoyancy predicted enjoyment in English speaking learning. Grounded in the control-value theory (Pekrun, 2006), achievement emotions arise from control and value appraisals. Specifically, when learners can control achievement activities (behaviors and outcomes) and recognize their value, achievement emotions are elicited. In this study, learners with higher academic buoyancy were better equipped to handle setbacks, fostering more positive control appraisals and maintaining more value appraisals in English speaking learning. This dual enhancement of control and value appraisals significantly increased enjoyment, a key positive achievement emotion (Xu and Wang, 2024). This finding further corroborates (Wang and Hui 2024), who identified a positive relationship between academic buoyancy and enjoyment in online learning (β = 0.307, *p* < 0.001), though the predictive coefficient in this study was slightly lower. Combined with the examination of participants' English speaking learning enjoyment in this study earlier, the smaller effect size suggested that teacher support and peer interaction were equally critical sources of learning enjoyment, alongside the ability to overcome challenges, in the context of spoken English learning.

Second, English speaking learning enjoyment was significantly and positively related to academic engagement (β = 0.393, *p* < 0.001). Learners who experienced higher enjoyment, marked by positive emotional experiences, supportive teacher interactions, and joyful classroom environments, as measured by the English Speaking Learning Enjoyment Scale, showed increased energy, passion, and immersion in English speaking learning. This was reflected in items on the English Speaking Learning Engagement Scale, such as “I am filled with energy in English speaking learning,” “When I am studying spoken English, I tend to forget about everything else around me,” and “As soon as I wake up in the morning, I look forward to practicing spoken English that day.” This finding aligns with (Aubrey 2025), who verified that heightened enjoyment during spoken tasks motivated EFL learners to think and complete established content. This finding could be explained by the broaden-and-build theory (Fredrickson, 2003), suggesting that positive emotions could expand learners' immediate thought-action repertoires, increasing their academic engagement (Yang and Liang, 2025). Meanwhile, it also corroborates (Yang and Liang 2025), demonstrating that enjoyment made a significant and positive effect on student engagement in the general EFL context (β = 0.65, *p* < 0.001). However, compared to their findings, enjoyment in this study had a weaker predictive effect on engagement, suggesting that its influence on academic engagement in English speaking learning was relatively limited.

Therefore, learners with higher academic buoyancy in English speaking learning possessed enhanced stability in learning and psychological capacities. This stability, in turn, fostered their greater perception of enjoyment, motivating them to invest more time and effort. Consequently, promoting enjoyment within the learning environment is crucial for enhancing engagement and academic performance in English speaking learning.

### 5.3 The moderating effects of English speaking learning anxiety (ESLA)

Further, this study elucidated the significant moderating roles of anxiety between academic buoyancy and both enjoyment and engagement in English speaking learning (β = −0.105, *p* < 0.01; 95% CI = −0.167 to −0.044 and β = −0.101, *p* < 0.05; 95% CI = −0.187 to −0.015). Specifically, ESLA significantly influenced how ESLB predicted ESLE and ESLEG. This extends previous research and corroborates the interactive effect of anxiety and buoyancy in academic contexts, as demonstrated by (Putwain et al. 2022), who revealed that academic buoyancy moderated the association between anxiety and test performance. Moreover, in English speaking learning, enjoyment and engagement significantly predict performance (Peng and Wang, 2024; Wang et al., 2024). Consequently, the interactive impact of academic buoyancy and anxiety on enjoyment and engagement in this context is logical. Additionally, when ESLA was low, ESLB most strongly predicted ESLE and ESLEG. Learners with lower anxiety, characterized by reduced nervousness and greater confidence in a spoken English class, as indicated by their disagreement with items in the English Speaking Learning Anxiety Scale, such as “Even if I am well-prepared for the spoken English class, I still feel nervous,” and “I always think that my classmates speak English better than me,” exhibited a stronger predictive effect of academic buoyancy—marked by the ability to effectively manage academic stress related to spoken English learning and to maintain positive attitudes, as assessed by the English Speaking Learning Buoyancy Scale—on both enjoyment and engagement. Specifically, these learners showed greater positive emotional experiences and reported more supportive classroom interactions, as measured by the English Speaking Learning Enjoyment Scale. They also demonstrated heightened energy, dedication, and absorption in their learning, as assessed by the English Speaking Learning Engagement Scale. This finding aligns with the established negative relationship between anxiety and both enjoyment and engagement among EFL learners (Wang and Li, 2022; Wang et al., 2023), explaining the negative moderating effects of ESLA in this research.

Although the moderating role of anxiety in the relationship between enjoyment and engagement was not statistically significant in this study (β = 0.009, *p* = 0.871), a positive moderating effect was observed, consistent with the positive but non-significant interplay of enjoyment and anxiety on behavioral engagement (β = 0.07, *p*>0.05) (Wang et al., 2025). This suggests that anxiety may partially moderate the relationship between academic buoyancy and academic engagement through the mediation of enjoyment in English speaking learning. This differs from other two sub-paths and the traditional view that anxiety has a negative impact on academic engagement (Wang et al., 2023). The discrepancy may stem from the characteristics of the participants in this research. Participants here exhibited higher enjoyment (mean = 5.947) than anxiety (mean = 3.883). Thus, the positive effect of enjoyment may have mitigated anxiety's negative impact on engagement. Consequently, ESLA had a positive but insignificant moderating effect between ESLE and ESLEG.

In summary, these findings highlight the multifaceted moderating roles of anxiety among Chinese college students in English speaking learning. Anxiety exerted both positive and negative moderating effects on the three sub-paths from ESLB to ESLEG via ESLE. These interactions underscore anxiety's significance in English speaking learning. Learners should leverage the moderating effect of anxiety to promote academic buoyancy's predictive impact on speaking engagement.

## 6 Conclusion, implications, and limitations

This study fills the theoretical gap in prior research by exploring the mechanisms among academic buoyancy, engagement, and foreign language learning emotions (enjoyment and anxiety) in the context of Chinese college students' English speaking learning. The findings reveal the complex interplay between academic buoyancy and these emotions in shaping engagement and offer valuable insights for future Chinese college English speaking instruction.

First, the direct predictive effect of ESLB on ESLEG suggests that teachers should focus on developing students' ability to overcome challenges and maintain a stable mentality. Specifically, teachers could provide emotional support and coping strategies to help students manage setbacks and pressure in spoken English learning. For example, teachers can design a self-reflection table for students to use in spoken English classes. During class, students record specific challenges they face (e.g., struggling with pronunciation or feeling nervous speaking publicly). After class, they document coping strategies, such as practicing difficult phrases or using positive self-talk (e.g., “It's okay to make mistakes”). Teachers review these tables, providing constructive feedback and offering emotional support through encouraging comments or brief check-ins. This approach enables students to gradually develop the ability to overcome setbacks and the mental stability by improving their hope for English speaking learning (Satici et al., 2024).

Second, the multifaceted roles of foreign language learning emotions indicate that teachers should enhance the positive effect of ESLE while flexibly leveraging the moderating effect of ESLA. Specifically, teachers should create a supportive English speaking learning environment by maintaining warm and patient attitudes, facilitating collaborative learning groups, and promoting a classroom atmosphere filled with laughter. Teachers can guide students to perform comedic scenes from classic movies or cartoons in small learning groups, fostering a humorous and engaging classroom atmosphere. This activity strengthens peer collaboration and group cohesion as students work together to prepare and perform. Additionally, it sparks students' imagination by encouraging creative interpretation of roles and dialogue, enhancing both enjoyment and confidence in spoken English.

Additionally, the complex moderating role of anxiety cannot be ignored. Before formal speaking instruction begins, teachers should assess students' original anxiety levels and identify their sources. If the anxiety is manageable, appropriate spoken English assessments can motivate students to improve. For students with lower spoken English proficiency, teachers can record individual speaking performances of these students, allowing students to review and practice repeatedly while correcting mistakes with guided feedback. For students with higher proficiency, teachers can encourage these students to speak confidently and loudly in class, followed by peer evaluation and constructive feedback. However, if students' anxiety is high, teachers should focus on mitigating anxiety and fostering a more relaxing learning environment by offering direct emotional support, incorporating humorous elements, and promoting self-praise and peer encouragement to enhance students' self-confidence (Demirdöken and Okur, 2023). Totally, by addressing the unique needs of diverse learners, teachers can simultaneously develop students' ability to overcome speaking challenges, improve students' enjoyment with humorous elements, and flexibly mitigate anxiety's negative effects to enhance students' engagement and performance in English speaking classes.

While this study provides valuable insights, some limitations should be acknowledged. The primary limitation pertains to the participant sample. The sample was exclusively drawn from students at two universities, and the sample size was not sufficiently large. This constraint limits the study's ability to capture the diversity of contexts and experiences in English speaking learning. Consequently, the generalizability of the findings may be restricted. Additionally, the complex path relationships among variables in this study, combined with the small sample size, may have contributed to an inflated SRMR value in the structural equation model (SRMR = 0.103 >0.08). Although the overall model fit is acceptable when considering other fit indices collectively (χ^2^/df = 2.135 < 3, RMSEA = 0.068, CFI = 0.966>0.95, TLI = 0.957>0.95) based on the two-index presentation strategy (Hu and Bentler, 1999), future studies should consider expanding the participant pool to mitigate the limitations associated with the small sample size. Specifically, future studies should encompass a wider range of universities, from top-tier to lower-tier institutions, and include students with diverse levels of English proficiency, and the SEM should be tested across different English proficiency levels. Such diversities would improve the robustness and the applicability of the findings across different learning environments and learner profiles. Fundamentally, this approach would strengthen the model's explanatory and predictive power. Additionally, participants in this study were drawn from diverse learning stages from undergraduates to doctoral candidates, resulting in variations in learning contexts and psychological cognition. Future research should account for these differences and conduct more targeted analyses of English-speaking learning.

Another limitation is related to the study's cross-sectional design. Although the present study reveals the relationships among academic buoyancy, engagement, enjoyment, and anxiety in the context of English speaking learning, the results are based on a single moment. This limits the capacity to draw conclusions about the dynamic and evolving nature of these variables over time. Therefore, future research should develop longitudinal studies to attain a more comprehensive understanding of the underlying mechanisms among these variables. Longitudinal designs would allow researchers to capture the temporal dynamics and potential causal relationships. For instance, future research could explore whether academic buoyancy influences enjoyment or engagement over time, when the moderating role of anxiety between enjoyment and engagement becomes more pronounced, or whether the direction of the relationships among these variables might reverse. Such investigations would provide a more comprehensive understanding of how academic buoyancy, engagement, and foreign language learning emotions interact within the context of English speaking learning.

## Data Availability

The raw data supporting the conclusions of this article will be made available by the authors, without undue reservation.
